# Interhemispheric Facilitatory Effect of High-Frequency rTMS: Perspective from Intracortical Facilitation and Inhibition

**DOI:** 10.3390/brainsci12080970

**Published:** 2022-07-23

**Authors:** Dongting Tian, Shin-Ichi Izumi

**Affiliations:** 1 Department of Physical Medicine and Rehabilitation, Graduate School of Medicine, Tohoku University, 2-1 Seiryo-cho, Aoba-ku, Sendai 980-8575, Japan; tian.dongting.p1@dc.tohoku.ac.jp; 2 Graduate School of Biomedical Engineering, Tohoku University, 2-1 Seiryo-cho, Aoba-ku, Sendai 980-8575, Japan

**Keywords:** interhemispheric facilitation, intracortical facilitation, intracortical inhibition, motor cortex, transcranial magnetic stimulation, voluntary movement

## Abstract

The activity of excitatory and inhibitory neural circuits in the motor cortex can be probed and modified by transcranial magnetic stimulation (TMS) and repetitive TMS (rTMS), noninvasively. At present, not only has a consensus regarding the interhemispheric effect of high frequency rTMS not been reached, but the attributes of these TMS-related circuits are also poorly understood. To address this question comprehensively, we integrated a single- and paired-pulse TMS evaluation with excitatory 20-Hz rTMS intervention in order to probe the interhemispheric effect on the intracortical circuits by high-frequency rTMS. In the rest state, after 20-Hz rTMS, a significant increase of single-pulse MEP and paired-pulse intracortical facilitation (ICF) in the non-stimulated hemisphere was observed with good test–retest reliability. Intracortical inhibition (measured by the cortical silent period) in the unstimulated hemisphere also increased after rTMS. No significant time–course change was observed in the sham-rTMS group. The results provide the evidence that 20-Hz rTMS induced a reliable interhemispheric facilitatory effect. Findings from the present study suggest that the glutamatergic facilitatory system and the GABAergic inhibitory system may vary synchronously.

## 1. Introduction

Neuromodulation, as a group of techniques by which activities of the nervous system are directly modulated, is gaining increasing popularity and acceptance as an important treatment for various neurological disorders (e.g., major depression, epilepsy, stroke, spinal cord injury, traumatic brain injury, etc.) [1,2,3,4]. By virtue of non-invasive brain stimulation (NIBS), not only can the impaired neural function be modulated in neurological patients, but the neural processes and network connectivity in the human brain can also be unveiled [5]. Transcranial magnetic stimulation (TMS) has been considered the only non-invasive approach able to both modulate and probe neuroactivity and neuroplasticity [6,7,8]. By applying single- and paired-pulse TMS to the primary motor cortex (M1), corticospinal excitability, as well as the activity of cortical facilitatory and inhibitory circuits, can be revealed by the amplitude of motor evoked potential (MEP) [7,9]. In particular, intracortical inhibition (ICI) can be probed not only with paired-pulse TMS, but also single-pulse TMS during voluntary muscle activation as the cortical silent period (CSP). CSP is defined as the temporary suppression of ongoing muscle activity after TMS-induced MEP. A longer suppression (silent period) indicates a stronger ICI. It has been concluded that CSP reflects the GABAergic ICI, with a CSP shorter than 100 ms reflecting the GABA_A_-mediated inhibition, while a CSP longer than 100 ms mainly reflects the GABA_B_-mediated ICI [9]. However, intracortical facilitation is usually assessed only using paired-pulse TMS (for a review, see [9]). Paired-pulse ICF consists of short interval ICF (SICF) and long-interval ICF (LICF) [10,11]. SICF is considered to reflect the cortical-mediated I-wave facilitation [12], while LICF may result from neural populations other than those generating the I-waves, as the two phenomena differ in ISI and pulse intensity [13]. However, not only do the attributes of SICF and LICF phenomena in rest and during voluntary muscle activation remain obscure, consensus on the physiological processes of ICF (cortical and subcortical) and their interaction with other cortical circuits also remains incomplete [14,15].

Apart from probing cortical activity, applying TMS repetitively at a certain frequency (i.e., repetitive TMS, rTMS) substantially modulates cortical excitability by inducing neuroplasticity. It has been widely proven that high-frequency (≥3 Hz) rTMS increases the cortical excitability of the stimulated hemisphere, whereas low-frequency rTMS (≤1 Hz) decreases it [9]. However, the effect of rTMS on the unstimulated hemisphere varies greatly, with different outcomes reported in both high- and low-frequency rTMS protocols (for review see [16]). This modulation can be referred to as an alteration of the cortical and subcortical excitability in the contralateral (unstimulated) M1, possibly through the interhemispheric pathways, including interhemispheric inhibition (IHI) and interhemispheric facilitation (IHF) acting upon the intracortical inhibitory and facilitatory circuits [17,18].

Currently, several important questions regarding TMS remain to be answered. First, consensus on the interhemispheric effect of excitatory rTMS and its reliability has not been reached, which demands systematic investigation. Second, the relationship between cortical facilitation and inhibition is also pending further investigation, owing to major difficulties in correlating neurophysiological phenomena (observed by TMS, etc.) with metabolic processes (measured using magnetic resonance spectroscopy, etc.) [19,20,21]. To answer these questions, we devised a comprehensive TMS protocol in the present study to explore: (1) the modulation of SICF and LICF by contralateral excitatory rTMS, and (2) the attributes of glutamatergic intracortical facilitation (SICF and LICF) under the influence of rTMS and voluntary drive. For the interaction between ICF and ICI, we sought to infer this relationship by analysing the interaction between ICF and cortical silent period (CSP, indicating the GABAergic ICI). We assumed that: (1) excitatory rTMS can reliably potentiate single-pulse MEP, SICF, and LICF in the non-stimulated hemisphere when assessed both in the rest state and during voluntary movement; (2) the modulation outcome differs between SICF and LICF; and (3) the relationship between the glutamatergic and GABAergic neuronal circuits can be revealed in the modulation of ICF and CSP.

## 2. Materials and Methods

### 2.1. Ethical Approval

The experiment protocol was approved by the Ethics Committee of Tohoku University Graduate School of Medicine (Protocol Identification Number: 2021-1-919) and conducted in accordance with the Declaration of Helsinki.

### 2.2. Participants

Forty-two healthy adults participated in the experiment and were randomly assigned as the real rTMS (twenty-two healthy adults, nine males, aged 26.8 ± 3.34 years) and age-matched sham-rTMS (twenty healthy adults, six males, aged 27.5 ± 4.62 years) groups. Written informed consent was obtained prior to the experiment. All participants were right-handed according to the Edinburgh Handedness Inventory [22]. None of the participants reported a history or current signs of neurological or musculoskeletal impairment. A questionnaire for TMS and rTMS was used to perform screening for TMS contraindications [23,24]. Sample size was selected based on previous rTMS studies [18,25] and an a-priori power analysis using the GPower 3.0 software (http://www.psycho.uni-duesseldorf.de/abteilungen/aap/gpower3/, accessed on 2 March, 2021). The alpha and beta errors were set as α = 0.05 and β = 0.2 with a medium effect size of f = 0.25. The calculation resulted in 17 subjects for each group.

### 2.3. Protocol

**Figure 1:**
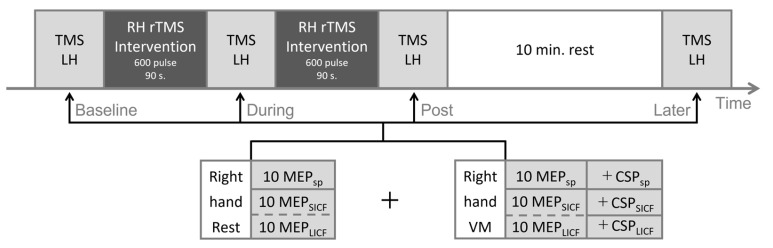
Experimental protocol. Repetitive TMS intervention (real or sham, dark grey) was applied in two sessions with 600 pulses each. TMS measurement (light grey) was performed at four timepoints throughout the experiment. The order of SICF and LICF measurements was randomized for each timepoint, as shown in dashed lines. LH = left hemisphere; RH = right hemisphere; VM = voluntary movement; MEP = motor evoked potential; SICF = short-interval intracortical facilitation; LICF = long-interval intracortical facilitation; CSP = cortical silent period; sp = single-pulse.

Participants of the real rTMS group underwent the comprehensive TMS experimental protocol in Figure 1 for three identical sessions, taking place during three consecutive days. In the rTMS intervention, cortical excitability of the right hemisphere was upregulated by 20-Hz rTMS. TMS measurement was performed at four time points (Baseline, During, Post and Later) across the experiment. In the rest condition, we measured single-pulse unconditioned MEP (MEP_sp_) with single-pulse TMS, and MEP_SICF_, MEP_LICF_ with paired-pulse TMS during the complete rest of the hand, which was confirmed by real-time electromyography (EMG). The same measurements were also performed separately in the context of voluntary movement (VM). For VM, we adopted slight isometric thumb abduction (i.e., isometric contraction of the abductor pollicis brevis, APB) to avoid possible involvement of the ipsilateral M1 as like crossed facilitation [26,27]. Specifically, participants were first instructed to perform maximum thumb abduction to produce a maximum voluntary contraction (100% MVC). Then, participants were instructed to slightly stretch from the inside a circular elastic band of 1-cm width using the interphalangeal (IP) joint of their thumb and the proximal interphalangeal (PIP) joint of their index finger, producing a 20 ± 10% MVC (approximately 20% of the amplitude of 100% MVC EMG) isometric contraction of the right APB. Throughout VM measurements, participants were not presented with visual real-time EMG, but received vocal feedback from the experimenter (who constantly monitored the real-time EMG) to maintain optimal muscle output.

For the sham-rTMS group, participants underwent the protocol in Figure 1 for only one session (one day). In the sham rTMS intervention, 20-Hz sham-rTMS was applied to the right hemisphere. The rTMS coil was positioned perpendicular to the cranium over the right M1. The rTMS stimulation intensity and TMS evaluation was conducted in accordance with the real rTMS group.

The experiment was carried out in a quiet, well-lit laboratory. During the experiment, participants were comfortably seated in an armchair with their arms resting on the armrest. Earplugs were used to avoid auditory influence by TMS. Given that attention can affect MEP size and rTMS modulation [28,29], participants were instructed to fixate on a printed fixation cross (5 × 5 cm) set 50 cm away from the front during the entire experiment.

### 2.4. TMS Parameters

#### 2.4.1. TMS Equipment and Basic Configuration

Single- and paired-pulse TMS were delivered to the left hemisphere through a figure-of-eight coil (70-mm double coil MAG-9925-00) connected to a pair of Magstim 200^2^ via a Bistim module (Magstim Co., Whitland, UK). The coil was oriented at an angle 45° to the midsagittal plane, with the coil handle pointing backwards to induce posterior-to-anterior current flow. Repetitive TMS (TMS intervention) was delivered to the right hemisphere using Magstim Rapid^2^ (Magstim Co., Whitland, UK) with a figure-of-eight coil (D70^2^ Coil), following the same coil orientation as the TMS measurement.

All TMS and rTMS were delivered to the M1 representational area of APB (i.e., the APB-hotspot, at the location where a suprathreshold single-pulse TMS evoked MEP with maximum amplitude in the APB muscle [30]), marked with a pen over the scalp. The TMS coil was held by an articulated mechanical arm (Manfrotto 244, VitecGroup, Cassola, Italy), with the coil junction centre placed tangentially over the APB-hotspot. TMS measurement was automatically executed by customised MATLAB 2021a (The MathWorks, Inc., Natick, MA, USA) scripts with the MAGIC toolbox [31].

#### 2.4.2. Measurement: Single-Pulse TMS

Prior to the experiment, the bilateral resting motor threshold (rMT) of each participant was determined using single-pulse TMS [32]. Based on the rMT, TMS intensity (% Maximum stimulator output, MSO) eliciting MEPs with 0.5- to 1-mV peak-to-peak amplitude (0.5–1-mV stimulation intensity, SI_0.5–1 mV_) of the right APB was also determined by gradually increasing the stimulus intensity (by 1%MSO) from rMT until 10 consecutive MEPs of optimal amplitude were elicited [33].

In the experiment, MEP_sp_ (rest and VM) was measured using the SI_0.5–1 mV_ TMS pulse. The stimulation interval for MEP_sp_ was set as 5 s [34]. Abundant evidence exists that 20-Hz rTMS can substantially increase MEP amplitude in the stimulated hemisphere, therefore we did not assess the MEP in the stimulated hemisphere according to the established consensus.

#### 2.4.3. Measurement: Paired-Pulse TMS

Paired-pulse TMS assessing SICF was given at S1 = SI_0.5–1 mV_ and S2 = 100% rMT. The ISI of SICF was set to 1.5 ms, corresponding to the ISI that elicits the clearest facilitatory I1-wave [12,35]. LICF was assessed at S1 = 70% rMT and S2 = SI_0.5–1 mV_ with 10 ms ISI [10,36]. To avoid possible carryover effect [37,38], SICF and LICF trials were applied every 10 s. The order of SICF and LICF measurements was randomised to prevent order effects.

#### 2.4.4. Intervention: Repetitive TMS

20-Hz rTMS intervention was applied for two 90-s sessions (Figure 1) with the stimulation intensity set as 70% rMT. Each train of stimulation consisted of 2-s stimulation of 40 pulses and 4-s inter-trial interval (ITI) [24,39,40]. During the rTMS session, participants were instructed to rest their arms completely. EMG activity of both APBs was constantly monitored by the experimenter to ensure no MEP was induced by subthreshold rTMS.

### 2.5. EMG Recording

EMG from bilateral APBs was recorded throughout the TMS evaluation and was monitored during rTMS intervention. The recorded EMG data was stored for offline analysis. Disposable surface electrodes (Ambu Blue Sensor N, N-00-S/25, Ambu A/S, Ballerup, Denmark) were placed over the APB muscle in a lengthwise belly–belly montage [41], with reference electrodes placed over the ulnar styloid process. Surface EMG signals were recorded with a bio-amplifier (MEG-6116 M, Nihon-kohden, Tokyo, Japan) connected to a PowerLab 16/35 device and processed using the LabChart Pro 8.0 software (AD Instruments Inc., Dunedin, New Zealand). Raw EMG signals were digitised at a sampling frequency of 10 kHz, amplified 1000×, and filtered within 20–450 Hz.

The time zone of all EMG analyses was set from 500 ms before TMS to 2000 ms after TMS. CSP was automatically calculated as the duration between the onset of MEP and restoration of pre-MEP average EMG amplitude (500 ms prior to TMS pulse) in VM [42].

### 2.6. Statistics

For the real rTMS group, the intrasubject difference of baseline MEP_sp_, MEP_SICF_ and MEP_LICF_ amplitudes from the 3-day repetitive measurement sessions was assessed using repeated measures analysis of variance (RMANOVA). The average EMG amplitude 500 ms prior to TMS was also processed using one-way ANOVA for intrasubject difference analysis, with DAY set as the independent variable.

Time-course modulation of all MEP_sp_, MEP_SICF_ and MEP_LICF_ amplitudes and CSP_sp_, CSP_SICF_ and CSP_LICF_ duration (VM only) contralateral to rTMS in the 3-day experiment was analysed using repeated-measures ANOVA (RMANOVA), with TIME (Baseline, During, Post0, Post10) set as the within-subject factor. The post hoc test of Bonferroni correction was performed for significant results or tendencies. To exclude the effects of baseline MEP_sp_ interindividual variability, we further normalised the outcomes of MEP_SICF_ and MEP_LICF_ by converting all data into a percentage of baseline MEP_sp_ values as the ratio of SICF and LICF and included the ratio in the statistical analyses [38,43]. All time-course modulation analyses were performed separately in rest and VM, to address the effects of muscle activation throughout the experiment.

In light of the effects of different paradigms, all MEP amplitude and CSP duration of single-pulse, SICF and LICF paradigms in the 3-day experiment were also analysed using one-way ANOVA with the independent variable PARADIGM (single-pulse, SICF and LICF). 

To determine the test–retest reliability of the measured parameters in the rest state and VM, intraclass correlation coefficients (ICCs) of all parameters were calculated based on the intrasubject data measured during the 3-day repetitive measurement. We calculated ICC based on a single-rater, one-way random effect for the absolute agreement model (i.e., ICC(1, 3)) [44].

For the sham-rTMS group, the baseline MEP_sp_ amplitude was compared with the average of the 3-day experiment baseline MEP_sp_ of the real rTMS group, using an independent two-sample Student’s *t*-test. Time-course modulation of all parameters (including the SICF and LICF ratio) was analysed using RMANOVA with Bonferroni corrections as in the real rTMS group. Inter-paradigm difference of the paradigms was also assessed using the same statistic method as the real rTMS group.

Statistical significance was accepted at *p* < 0.05. The IBM SPSS Statistics v.26 software (IBM Corp., Armonk, NY, USA) was used to perform all statistical analyses. Figures were generated using customized MATLAB scripts.

## 3. Results

The experiment protocol was well tolerated by all participants. However, in the real rTMS group, stable MEPs > 0.2 mV could not be evoked from one individual during the entire experiment, and another individual withdrew on the third day for personal reasons. Data from these two participants were excluded from all statistical analyses.

The rest motor threshold of the real rTMS group was 53.55 ± 6.84%MSO for the left hemisphere and 54.60 ± 6.76%MSO for the right hemisphere. For the sham-rTMS group, the rMT was 55.1 ± 7.33%MSO and 55.9 ± 6.55%MSO for the left and right hemispheres respectively. The intensity for SI_0.5–1 mV_ (left hemisphere only) was 64.60 ± 6.47%MSO and 66.45 ± 8.09%MSO for the real- and sham-rTMS group.

### 3.1. Baseline Measurements

For all TMS-measured variables in the real rTMS group, there was no significant baseline intrasubject difference in the 3-day repetitive measurement of all parameters (MEP amplitude and CSP duration, all *p* > 0.05, Supplementary Table S1). For the EMG activity measured 500 ms prior to TMS pulse in the VM condition, one-way ANOVA showed no significant intrasubject difference among the 3 days (F_2717_ = 0.691, *p* = 0.502), indicating no carry-over effects along the 3-day experiment sessions. There was no significant difference in the rest and VM baseline MEP_sp_ between the real rTMS and sham-rTMS groups (*n* = 20, rest *p* = 0.43, VM *p* = 0.12, independent two-sample Student’s *t*-test).

### 3.2. Interhemispheric ICF Modulation by rTMS

**Figure 2:**
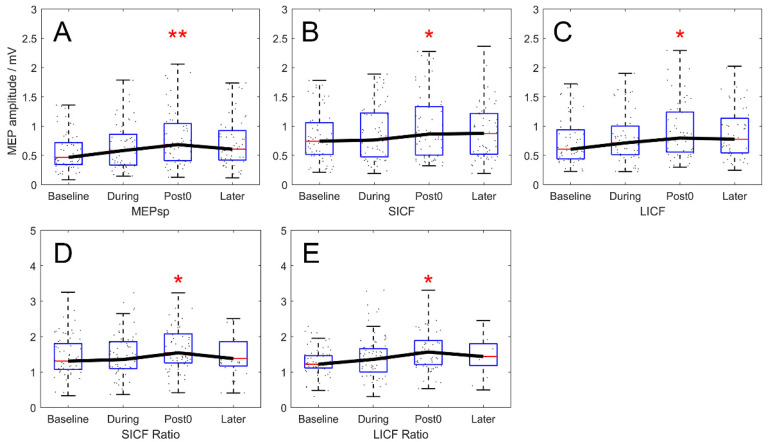
Chronological modulation of MEP_sp_, SICF and LICF in rest condition (real-rTMS group). (**A**–**C**) Outcomes expressed in MEP amplitude (mV). MEP amplitude of rest MEP_sp_, MEP_SICF_ and MEP_LICF_ increased significantly at the Post timepoint, compared to Baseline. (**D**,**E**) Outcomes expressed in ICF ratio normalized by baseline MEP_sp_ amplitude. Normalized SICF and LICF ratio also showed significant enhancement at the Post timepoint compared to Baseline. Boxes denote 25th and 75th percentile values, whiskers denote minimum and maximum values. Asterisks denote significant difference compared to the Baseline timepoint. * *p* < 0.05; ** *p* < 0.01.

MEP_sp_, MEP_SICF_ and MEP_LICF_ amplitudes measured in the rest state throughout the experiment (real-rTMS group) are shown in Figure 2. Difference between rest, MEP_sp_, MEP_SICF_ and MEP_LICF_ by TIME reached significance by RMANOVA (F_3,217_ = 4.830, 3.443, and 3.578; *p* = 0.003, 0.018 and 0.015 for MEP_sp_, MEP_SICF_ and MEP_LICF_). The post hoc test revealed significant increase in rest, MEP_sp_, MEP_SICF_ and MEP_LICF_ between the Baseline and Post time points (*p* = 0.002, 0.016 and 0.013 for MEP_sp_, MEP_SICF_ and MEP_LICF_, Figure 2A–C). Moreover, compared to that at baseline, the rest MEP_sp_ at the later timepoint demonstrated a tendency to increase, yet the post hoc test failed to reach statistical significance (*p* = 0.089). At the During timepoint, there was no significant chronological effect of any MEP parameter measured. In VM, no significant chronological modulation of MEP at any timepoint was shown (F_3,217_ = 0.461, 0.176, and 0.555; *p* = 0.710, 0.913 and 0.645 for MEP_sp_, MEP_SICF_ and MEP_LICF_).

After normalisation, along with the significant effect of TIME (F_3,217_ = 2.803 and 2.985; *p* = 0.041 and 0.032 for SICF and LICF ratio), the increase of the rest SICF and LICF ratios remained significant between the Baseline and Post timepoints and returned to baseline 10 min after rTMS (Baseline–Post: *p* = 0.045 for SICF ratio; *p* = 0.030 for LICF ratio. Baseline–Later: *p* = 0.571 for SICF ratio; *p* = 0.154 for LICF ratio; after correction; Figure 2D,E).

In the sham-rTMS group, no significant time-course modulation of all MEP amplitudes (including SICF and LICF ratio) was revealed (RMANOVA, *p* > 0.05, Supplementary Table S2).

### 3.3. Interhemispheric ICI Modulation by rTMS

**Figure 3:**
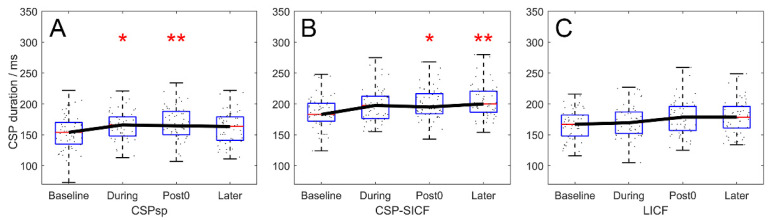
Chronological modulation of CSP duration by 20-Hz rTMS, in the real-rTMS group. (**A**) modulation of CSP_sp_. (**B**) modulation of CSP_SICF_. (**C**) modulation of CSP_LICF_. CSP_sp_ demonstrated significant prolongation at the During and Post timepoints, while CSP_SICF_ and CSP_SICF_ showed significant prolongation at the Post and Later timepoints. The prolongation of CSP_LICF_ was not significant. Boxes denote 25th and 75th percentile values, whiskers denote minimum and maximum values. Asterisks denote significant difference compared to the Baseline timepoint. * *p* < 0.05; ** *p* < 0.01.

CSP_sp_, CSP_SICF_, and CSP_LICF_ modulation throughout the experiment (real-rTMS group) is plotted in Figure 3. A significant effect of TIME was revealed in all three paradigms (F_3,217_ = 4.876, 4.928, and 3.011; *p* = 0.003, 0.002 and 0.031 for CSP_sp_, CSP_SICF_ and CSP_LICF_). The post hoc test showed that chronological modulation of CSP_sp_ demonstrated significant prolongation at the During and Post timepoint, and returned to baseline at the Later timepoint (Baseline–During, *p* = 0.017; Baseline–Post, *p* = 0.006; Baseline–Later, *p* = 1.000, Figure 3A), whereas the increase of CSP_SICF_ reached significance at the Post timepoint and demonstrated significant prolongation 10 min after rTMS (Baseline–Post, *p* = 0.021; Baseline–Later, *p* = 0.003, Figure 3B). However, despite the significant difference by TIME, after correction, the CSP_LICF_ only showed a tendency to increase (Baseline–Post, *p* = 0.069; Baseline–Later, *p* = 0.053, Figure 3C).

In the sham-rTMS group, no significant time-course modulation of all CSP durations was revealed (RMANOVA, *p* > 0.05, Supplementary Table S2).

### 3.4. Paradigm-Wise Effects of Rest and VM

**Figure 4:**
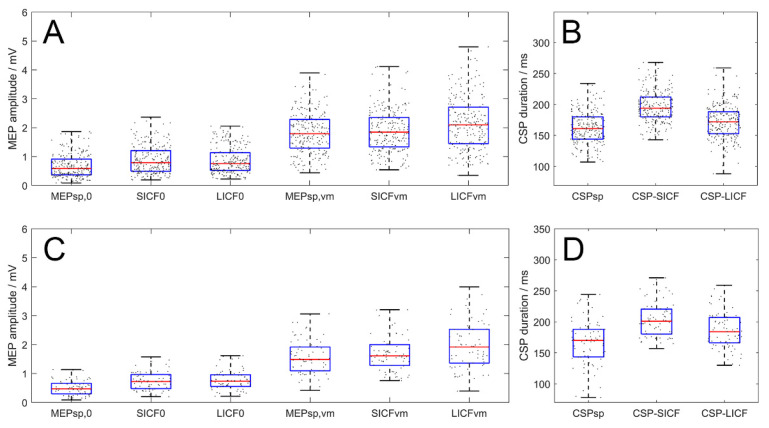
Paradigm-wise effects of MEP amplitude and CSP duration of all timepoints (real- and sham-rTMS group, upper and lower panel respectively). (**A**,**C**) Amplitude of MEP_sp_, MEP_SICF_ and MEP_LICF_. In VM condition, MEP_LICF_ demonstrated significant facilitatory effect while the facilitation of MEP_SICF_ was absent. (**B**,**D**) CSP duration in the three paradigms. CSP_SICF_ and CSP_LICF_ duration were longer than the CSP_sp_, with CSP_SICF_ significantly longer than CSP_LICF_. Boxes denote 25th and 75th percentile values, whiskers denote minimum and maximum values.

In the rest condition, the overall MEP_SICF_ and MEP_LICF_ were enhanced by 176.1 ± 89.2% and 173.60 ± 93.6% (real-rTMS group, *n* = 20 × 3 days, F_2717_ = 13.773, *p* < 0.001) compared to MEP_sp_, respectively. In VM, MEP modulation also reached statistical significance (F_2717_ = 6.682, *p* = 0.001), with MEP_LICF_ being significantly greater than MEP_sp_ (*p* = 0.002) and MEP_SICF_ (*p* = 0.015) as revealed by the *post hoc* test. However, in VM, the difference between MEP_sp_ and MEP_SICF_ amplitudes was not significant (*p* = 1.000, Figure 4A). In terms of CSP, the overall duration of CSP_SICF_ (200.32 ± 30.00 ms) was significantly longer than that of CSP_sp_ (165.94 ± 32.76 ms, *p* < 0.001) and CSP_LICF_ (174.33 ± 32.72 ms, *p* < 0.001). Additionally, the duration was also longer for CSP_LICF_ than for CSP_sp_ (*p* = 0.012, Figure 4B).

For the sham-rTMS group, in rest state, MEP_SICF_ and MEP_LICF_ were enhanced by 60.4 ± 65.8% and 63.7 ± 74.7% (*n* = 20, F_2717_ = 7.049, *p* = 0.001) compared to MEP_sp_, respectively. In VM, MEP modulation also reached statistical significance (F_2717_ = 5.620, *p* = 0.004), with MEP_LICF_ being significantly greater than MEP_sp_ (*p* = 0.003) as revealed by the post hoc test. However, in VM, the difference between MEP_sp_ and MEP_SICF_ amplitudes was not significant (*p* = 1.000). Meanwhile, the difference between MEP_SICF_ and MEP_LICF_ failed to reach statistical significance in the post hoc test (*p* = 0.146). In terms of CSP, one-way ANOVA revealed a significant effect of PARADIGM on the CSP duration (*n* = 20, F_2237_ = 17.732, *p* < 0.001). Post-hoc test revealed that the overall duration of CSP_SICF_ (199.46 ± 34.68 ms) was significantly longer than that of CSP_sp_ (167.01 ± 35.63 ms, *p* < 0.001) and CSP_LICF_ (183.11 ± 33.02 ms, *p* = 0.009). Similarly, the duration of CSP_LICF_ was also longer than CSP_sp_ (*p* = 0.010).

### 3.5. Parameter-Wise Test–Retest Reliability

MEP_sp_, MEP_SICF_ and MEP_LICF_ in the 3-day repetitive measurement (real-rTMS group) demonstrated good to excellent test–retest reliability in both rest and VM (ICC rest [95% confidence interval]: MEP_sp_ = 0.766 [0.661–0.842]; MEP_SICF_ = 0.839 [0.766–0.892]; MEP_LICF_ = 0.803 [0.714–0.867]; ICC VM: MEP_sp_ = 0.872 [0.815–0.914]; MEP_SICF_ = 0.838 [0.765–0.891]; MEP_LICF_ = 0.868 [0.809–0.911], all *p* < 0.001). ICC of CSP_sp_ and CSP_SICF_ resulted in moderate to good intra-rater reproducibility, whereas ICC of CSP_LICF_ was poor, different from that of the other two paradigms (ICC CSP_sp_ = 0.715 [0.587–0.808]; CSP_SICF_ = 0.762 [0.655–0.840], *p* < 0.001; ICC of CSP_LICF_ = 0.454 [0.209–0.633], *p* = 0.001).

## 4. Discussion

The present study provides three main findings. First, 20-Hz rTMS induced interhemispheric facilitatory effect, potentiating rest MEP_sp_, MEP_SICF_ and MEP_LICF_ in the unstimulated hemisphere with good test–retest reliability. Second, attribute differences coexisted between SICF and LICF paradigms. Third, ICI also increased in the presence of IHF, with different modulation among the three paradigms of single-pulse TMS, SICF and LICF.

### 4.1. Interhemispheric Facilitatory Effects of 20-Hz rTMS

The interhemispheric effects of excitatory high-frequency rTMS remains controversial (for a review, see [18]) when assessed with TMS. However, studies using fMRI [45,46,47,48], fluorodeoxyglucose-positron emission tomography (FDG-PET) [49], functional near-infrared spectroscopy (fNIRS) [50] and electroencephalogram (EEG) [51,52] have reported that excitatory rTMS induced cortical activation in both the stimulated and non-stimulated hemisphere, even though the neuroplastic effect and its persistency varied by stimulation frequency, dose and pattern. In the present study, we observed such IHF effects using TMS, showing as the MEP enhancement at the Post time points in the real-rTMS group (but not in the sham-rTMS group). Upon considering the result of the sham group, in which no time-course MEP modulation was shown, we expect the modulation to be induced by the rTMS intervention instead of the influence of environmental and other external factors. Given that callosal projections are excitatory and glutamatergic [53,54], this interhemispheric MEP_sp_, SICF and LICF facilitation may stem from the transcallosal glutamatergic pathway, indicating a possible contribution of the glutamate-mediated IHF. This result appears to be contradictory to the traditional IHI theory, according to which the excitation of one hemisphere should result in inhibition of the contralateral hemisphere. We believe that this IHF observation is essential to the understanding of the interhemispheric interaction mechanisms.

### 4.2. Modulation of ICF by 20-Hz rTMS

Despite the evidence of ipsilateral ICF increase after excitatory high-frequency rTMS [25,55,56], contralateral (interhemispheric) ICF modulation by excitatory rTMS remains controversial. Jung et al. [57] reported persistent LICF suppression and SICI enhancement in the unstimulated hemisphere after 20 trains (1000 pulses) of 10-Hz rTMS, along with an inhibition of MEP amplitude. On the other hand, Gorsler et al. [16] reported no SICI and LICF changes in the unstimulated hemisphere after 1800 pulses of 5-Hz rTMS, whereas 1800 pulses of 0.5-Hz rTMS decreased LICF contralateral to rTMS. Our results contradict the aforementioned evidence by presenting a significant increase in the amplitude and ratio of both SICF and LICF after 1200 pulses of 20-Hz rTMS. We attribute this incongruity to the expression of ICF. The majority of paired-pulse TMS studies have normalised the paired-pulse ICF and ICI amplitude into a percentage of the corresponding single-pulse MEP amplitude [58], which bears the risk of a ceiling effect when rTMS raises the amplitude of single-pulse MEP through long-term potentiation (LTP) [59,60]. The mechanisms of ICF also remain elusive at present, we therefore adopted the normalisation of baseline MEP_sp_ amplitude to exclude the individual variability while preserving possible rTMS-induced variation of the parameters. This increase in ICF indicates that the excitability of the ICF circuit was also potentiated by 20-Hz rTMS, in accordance with the potentiation of the MEP_sp_.

### 4.3. Modulation of ICI by 20-Hz rTMS

In the present study, the interhemispheric ICI modulation measured by CSP increased with the contralateral 20-Hz rTMS in all three paradigms, which is supported by the evidence for ipsilateral CSP increase after 20-Hz rTMS [61]. As post-rTMS (ipsilateral) CSP duration tends to increase with higher rTMS frequency [62,63], we propose that rTMS with higher frequency can summon additional inhibitory postsynaptic potentials (IPSPs, lasting approximately 200–300 ms, [64]) from the asynchronous GABA release by the inhibitory interneurons, with the pulse interval being shorter than the duration of the IPSPs. However, while the restoration of CSP_sp_ was in agreement with previous findings [61], we present for the first time that the CSP_SICF_ and CSP_LICF_ (albeit tendency only) prolongation outlasted CSP_sp_ for 10 min after rTMS. As the thresholds of ICI circuits are below rMT [9,12], we speculate that the low-threshold ICI circuits summoned by subthreshold rTMS were also activated by the subthreshold conditioning pulse of SICF and LICF, causing the persistent CSP prolongation.

### 4.4. Attributes of ICI and ICF

In the present study, significant differences were identified between the three TMS measurement paradigms. The results demonstrated that MEP_LICF_ was not suppressed by VM, in contrast with MEP_SICF_. As voluntary drive can suppress the effects of ICF and ICI when muscle output increases [65,66], it is intriguing that the facilitatory effects of LICF were not suppressed by VM in the present study. Moreover, CSP_SICF_ and CSP_LICF_ also differed in duration and rTMS-induced chronological modulation. The difference between CSP_SICF_ and CSP_sp_ was also found in the work of Kojima et al. [67], reporting longer CSP_SICF_ than CSP_sp_ under constant intensity of the suprathreshold test stimulus. However, in another study by Silbert et al. [68], no difference between CSP_SICF_ and the SICF-amplitude-matched CSP_sp_ was reported. From this, we infer that the difference between CSP_sp_ and CSP_SICF_ is merely due to the integrated stimulus intensity of the paired pulse. Interestingly, we observed that CSP_LICF_ was significantly shorter than CSP_SICF_, despite the integrated stimulation intensity similar to that of CSP_SICF_. In relation to CSP, previous triple-pulse studies showed that LICF was not influenced in the presence of LICI [69,70], along with the fact that I-waves were not affected by LICF [70], highlighting the neural difference between LICF and SICF. Therefore, we speculate that LICF, although sharing a common neural basis with SICF, may bypass the inhibitory system to some extent, with its neural mechanisms differing from those of SICF.

### 4.5. Limitation

It is important to note that our study is limited in four aspects. First, the paired-pulse IHF was not assessed, thus a comparison of interhemispheric ICF modulation and paired-pulse IHF cannot be completed to explore the specific contribution of IHF. Second, IHI was also not assessed, which, to some extent, can bring uncertainty to the underlying process of interhemispheric interaction that caused the excitatory interhemispheric effect of 20-Hz rTMS. Third, since we adopted isometric contraction of the hand muscle, the modulation in movement initiation and task conditions demands further exploration. As IHI is lifted prior to movement initiation [71], the possibility exists that integrating the TMS intervention with the timing of movement may yield better modulation effects. Fourth, although we presented the main IHF results with good test–retest reproducibility, the influence of the nature variability of MEP due to both physiological and environmental factors is unignorable [9]. Since cortical excitability and intracortical circuitry can be alternatively probed with novel protocols such as high-resolution cerebral blood volume (CBV-fMRI) [72] and quantitative EEG [73], we believe that further evidence on the attributes of rTMS-induced IHF would be of great importance, as the presence of IHF possesses a great potential to both the understanding of the circuit wiring of the human brain and the promotion of neurorehabilitation.

## 5. Conclusions

In summary, the present study provides evidence that 1200 pulses of 20-Hz rTMS applied to the M1 can induce an interhemispheric facilitatory effect, increasing the MEP responses to single-pulse TMS, paired-pulse short- and long-interval intracortical facilitation in the unstimulated hemisphere with sufficient test–retest reproducibility. This result shows the possibility of an interhemispheric facilitation mechanism other than the traditional interhemispheric inhibition theory. We also propose that the ICF and ICI may vary synchronously, opposing the generally accepted concept of ‘antagonistically.’ In the mechanistic aspect, for the first time, we discover that LICF is less influenced by the intracortical inhibitory circuits compared to SICF. Although the mechanisms and brain networks underlying TMS still warrant further exploration, we suggest that interhemispheric facilitation would be a novel way to promote neurorehabilitation outcomes, as well as TMS technological innovations, to a higher level.

## Data Availability

The data presented in this study are available on request from the corresponding author.

## Supplementary Materials

The following supporting information can be downloaded at: https://www.mdpi.com/article/10.3390/brainsci12080970/s1, Table S1: Mean (SD) of the 3-day baseline MEP amplitude and CSP duration; Table S2: Mean (SD) of the chronological modulation of MEP amplitude and CSP duration (sham-rTMS group).
