# Supplementary material for: Interhemispheric Facilitatory Effect of High-Frequency rTMS: Perspective from Intracortical Facilitation and Inhibition
Source: Brain Sci. 2022 Jul 23;12(8):970. doi: 10.3390/brainsci12080970 (PMC9332419; doi:10.3390/brainsci12080970)
Supplement: Supplementary file 1 [file brainsci-12-00970-s001.zip › brainsci-1825128-supplementary.pdf]

Condition	Parameter	Day1	Day2	Day3	$F_{2,57}$	*p*
Rest	MEP_{sp} / mV	0.677(0.44)	0.653(0.43)	0.589(0.36)	0.244	0.784
	MEP_{SICF} / mV	0.993(0.49)	0.774(0.46)	0.885(0.53)	0.972	0.384
	MEP_{LICF} / mV	0.949(0.53)	0.753(0.48)	0.861(0.51)	0.787	0.460
V.M.	MEP_{sp} / mV	1.917(1.09)	2.098(1.16)	1.998(1.15)	0.129	0.879
	MEP_{SICF} / mV	1.995(0.95)	2.251(1.24)	2.063(1.21)	0.272	0.763
	MEP_{LICF} / mV	2.208(1.17)	2.500(1.61)	2.337(1.34)	0.224	0.800
CSP	CSP_{sp} / ms	154.95(25.34)	160.8(32.69)	156.85(35.30)	0.181	0.835
	CSP_{SICF} / ms	194.70(33.26)	189.50(29.89)	193.80(34.37)	0.146	0.865
	CSP_{LICF} / ms	169.00(27.52)	158.15(31.41)	169.25(34.50)	0.821	0.445

Supplementary Table 1. Mean (SD) of the 3-day baseline MEP amplitude and CSP duration. V.M. = voluntary movement; MEP = motor evoked potential; SICF = short interval intracortical facilitation; LICF = long interval intracortical facilitation; CSP = cortical silent period; sp = single pulse.

Condition	Parameter	Baseline	During	Post	Later	$F_{3,57}$	*p*
Rest	MEP_{sp} / mV	0.558(0.31)	0.665(0.64)	0.641(0.56)	0.553(0.34)	0.678	0.569
	MEP_{SICF} / mV	0.877(0.39)	0.808(0.37)	0.820(0.41)	0.738(0.32)	0.978	0.410
	MEP_{LICF} / mV	0.833(0.35)	0.857(0.54)	0.849(0.42)	0.779(0.32)	0.135	0.939
V.M.	MEP_{sp} / mV	1.559(0.62)	1.725(0.71)	1.706(0.76)	1.614(0.70)	1.025	0.388
	MEP_{SICF} / mV	1.758(0.84)	1.821(0.79)	1.907(0.75)	1.806(0.73)	0.785	0.507
	MEP_{LICF} / mV	2.003(1.02)	2.095(0.85)	2.164(1.15)	2.042(0.78)	0.632	0.597
CSP	CSP_{sp} / ms	160.50(42.50)	165.80(27.32)	172.90(34.35)	168.85(38.22)	1.232	0.307
	CSP_{SICF} / ms	192.70(30.50)	197.70(38.98)	208.85(32.10)	198.60(37.10)	1.917	0.137
	CSP_{LICF} / ms	182.60(26.97)	178.10(37.87)	188.95(34.28)	182.80(33.69)	0.410	0.746

Supplementary Table 2. Mean (SD) of the chronological modulation of MEP amplitude and CSP duration (sham-rTMS group). V.M. = voluntary movement; MEP = motor evoked potential; SICF = short interval intracortical facilitation; LICF = long interval intracortical facilitation; CSP = cortical silent period; sp = single pulse.
